# Hospitals and Unsold Newspapers: Lord Rowallan's Suggestion

**Published:** 1913-05-31

**Authors:** 

**Affiliations:** Chairman, The Victoria Infirmary of Glasgow. 26 Hans Place, S. W.


### THE EDITOR'S LETTER-BOX.

### Hospitals and Unsold Newspapers: Lord Rowallan's Suggestion.

*To the Editor of* The Hospital.

Sir,—A great number of illustrated weekly and other papers which might be of great value to the patients in our hospitals are unsold and returned to the publishers, and sold by them as waste paper.

Would it not be possible to form a small Advisory Committee from the Chapters, or others who take charge of the literature in some of our larger hospitals (in poorer neighbourhoods where supplies from other sources are insufficient), to take counsel together as to how this supply could be made available, and what papers it would be most desirable to get?

I have already had very encouraging answers in reply to telephoned inquiries to the *Illustrated London News* and *Graphic*, and I hear that the London Hospital alone could take a hundred copies of each of such publications.

I shall be glad to hear from anyone responsible for the literature of any of the large hospitals who would be prepared to meet others similarly interested.—Yours truly,

Rowallan,
Chairman, The Victoria Infirmary of Glasgow.

26 Hans Place, S.W., May 27, 1913.

[This letter is referred to in our Notes this week.—Ed., The Hospital.]

### The Comic History of British Nursing.

*To the Editor of* The Hospital.

